# Unraveling the effect of differential leadership on employee performance: Evidence from China

**DOI:** 10.3389/fpsyg.2023.1081073

**Published:** 2023-03-02

**Authors:** Ning Liu, Honglie Zhang, Jiang Zhou

**Affiliations:** ^1^Yunnan University of Finance and Economics, Kunming, Yunnan, China; ^2^International Business School, Yunnan University of Finance and Economics, Kunming, Yunnan, China

**Keywords:** differential leadership, perceived organizational support, individual-organizational fit, employee performance, intimacy energy

## Abstract

This paper examines how differential leadership influences employee performance *via* perceived organizational support and individual-organizational fit. The psychological contract theory was used to investigate how differential leadership influences the performance of employees. The information was obtained by means of surveys distributed to various types of Chinese firms. A total of 358 complete responses for statistical analysis were received and examined. A structural equation model was used, which we believed would be the most appropriate model for testing the proposed study model. The evidence suggests that differentiated leadership promotes employee performance. The association between leadership differences and employee performance is positively mediated by perceived organizational support. Individual-organizational fit lowers the positive effect of differential leadership on employee performance and reduces the positive effect of organizational support perception on employee performance. The results of this research contribute to a better understanding of Chinese culture’s differentiated interpersonal cordial leadership construct.

## Introduction

Differential leadership is a crucial factor influencing the growth of Chinese firms. Differential leadership, which is dissimilar to other leadership styles with Chinese cultural traits, highlights the impact of interpersonal cordiality on organizational productivity (Haibo, 2022). In differential leadership, the emotional closeness between leaders and employees influences the management of firms. In Chinese relationship society, differential leadership has evolved into a type of independent leadership. However, it has always been neglected by researchers. Not only has the outstanding performance of Chinese enterprises in the global market attracted a vast amount of attention from the international community, but it also drew out the unique leadership style of Chinese enterprises which in turn become the focus of attention (Lu et al., 2022). Being exempt from other leadership types with Chinese cultural characteristics, such as authoritarian and moral leadership, differential leadership emphasizes the role of interpersonal cordiality in organizational management (Chong, 2022). Authoritarian leaders strengthen their control over employees by enhancing their power status; thus, most employees are managed by command (Griep and Bankins, 2022). Ethical leaders manage and properly coordinate their employees by improving their personalities. The main objective of ethical leadership is to bring about spontaneous reverence and admiration among employees. Upon reviewing existing literature, it is of great importance to deeply explore the influence mechanism of this unique leadership style on the working state of employees. Interpersonal relationships will be regarded separately by relatives, distances, and cordiality in Chinese society. Moreover, decisions made by people about communicating and getting along with others are based on cordiality (Zhang et al., 2021). Differential leadership can be procured from the characteristics of interpersonal relationships.

Employee performance is explicitly associated with interpersonal relationships among colleagues in Chinese enterprises (Sahlmueller et al., 2022). Further, the interpersonal relationship is impacted by the degree of cordiality and status and characteristics of “differentiation” and “prioritization.” The treatment of employees by their leaders is influenced by their leadership style and the cordiality between the leader and his employees (Lee and Farh, 2019). Leaders will unintentionally treat employees within their vicinity differently because of the differential cordiality of relationships; then they try their best to find a balance between “differentiation” and “prioritization” (Lerutla and Steyn, 2022). We not only examine the impact of differential leadership on employee performance but also revise strategies for balancing the differences between coworkers. The paper investigates how differential leadership, in contradiction to the concept of fairness, affects employee performance. It will contribute to a more profound knowledge and practical investigation of leadership theory within the context of Chinese culture.

More recent studies on leadership styles in Chinese culture include moral, ethical, benevolent, and authoritarian leadership (Lopez-Cabarcos et al., 2021). Most studies pay more attention to how different leadership types affect employees’ organizational performance from theoretical and empirical perspectives. Louick and Muenks, 2021 talked about the direct influence and interactive impact of three kinds of paternalistic leadership (benevolent leadership, ethical leadership, and authoritarian leadership) on employees’ emotional experience of the “differentiation” and “prioritization” atmosphere. They reviewed its mediation effect on organizational citizenship behavior. Sorlie et al. (2022) outlines the main characteristics of differential leadership as “differentiation” and “prioritization,” which impact employee performance. Moral leadership, ethical leadership, and paternalistic leadership emerge from the internal cultural quality of leaders, while differential leadership closely monitors the frequently alternating external interpersonal network (Nassif et al., 2021). Differential leadership research has just begun, and theoretical and empirical studies still need to be enriched. Consistent with speculations about “differentiating” and “prioritizing” interpersonal social relations, this study organizes the theoretical origin of differential leadership thinking and conducts the comparative empirical inquiry, thus contributing to a deeper understanding of the concept of differential leadership.

Within the existing literature on the study of differential leadership, there is still room for improvement. The main way to account for this is to study only the impact of differential leadership on other variables, such as employee creativity, innovation behavior, and turnover intention, while at the same time sweeping aside the theoretical analysis of differential leadership (Kang and Han, 2021). Lerutla and Steyn (2022) inspected the mediating role of perceived identity between differential leadership and organizational citizenship behavior. These papers represent a specific aspect of employees’ behaviors; they have explored the mechanisms of influence of the individual and organizational behaviors under differential leadership and stimulated the development of differential leadership thinking. In terms of identifying the most influential pathway and selecting outcome variables, it must accurately measure the effect of organizational management used by differential leadership. Drawing on psychological contract theory, we capture employee performance as the outcome variable and initialize the mediating variable of perceived organizational support as well as the moderating variable of individual-organizational fit. This is because affective exposure to perceived organizational support is the overall assessment of the organizational atmosphere, and leadership style is the main driver of influence (Wang, 2021). Individual-organizational fit highlights how individuals cope with the organization, and employees themselves are the key influencers.

Organizational management in Chinese culture places an emphasis on cordial interpersonal relationships (Chong, 2022). It is imperative to identify leadership styles within Chinese culture and further enrich differential leadership theory. It is important to note that the intensity of cordiality in interpersonal relationships could be altered at any time during the same activity. These alterations are not binary transformations between “insiders” and “outsiders,” but are procedures of moderate progression in the degree of cordiality of the reciprocal relationships. Psychological contracts are not tangible contracts but use the influence of actual agreement. The purpose of the current study is to analyze the path of influence of differential leadership on employee performance under the mediating role of perceived organizational support and the moderating role of individual fit in the organization. Since we have analyzed the path of differential influence of leadership on employee performance under the mediating role of perceived organizational support as well as the moderating role of individual organizational fit, the recognition of leadership style in Chinese culture is helpful, as it further enriches the theory of differential leadership. The results of this study have practical guiding significance for improving employee performance in an organization with differential leadership.

## Theory and hypotheses

### Differential leadership and employee performance

“Psychological contract” was a term put forward by Professor Argyris, a famous American organizational psychologist, and was later perfected by Levinson and other researchers. Argyris defines a psychological contract as “a match between what the individual will give and what the organization wants to achieve, and what the organization will provide as a response to what the individual expects to achieve” (Argyris, 1957). The core content of psychological contract theory focuses on the psychological state of employees in the firm, involving three core constructs: job satisfaction, job involvement, and organizational commitment. Employee job satisfaction is the key to the management of the psychological contract of the firm (Davidescu et al., 2020). The goal of psychological contract management is to improve job satisfaction through emotional care for employees in order to achieve a strong sense of organizational belonging and a high degree of work engagement (Coyle-Shapiro et al., 2019). Kutaula et al. (2019) points out that the creation of a relational network with cordial emotions as the link is an effective method for improving the peaceful working environment of the organization. The famous Chinese social scientist Fei Xiaotong also realized this point and categorized the features of Chinese rural social relations as “differentiation” and “prioritization.” The original meanings of the differential cordiality schemas referred to people’s partiality inclinations that led to their differential judgment and processing of their environment. This principle or rule might develop differential relationships in human social relations (Louick and Muenks, 2021). In traditional Chinese culture in particular, the cordiality of interpersonal relationships profoundly influences people’s collective behavior, and direct influences the internal cooperative relationship of various organizations (Sorlie et al., 2022). Given this, differential relations arise under the action of various magnitudes and the nature of the relations. In organizational management, “differentiation” and “prioritization” styles are common among leaders, although they find it hard to explain these states themselves (Chong, 2022). Differentiation refers to the idea that individuals have diverse psychological perceptions of the world around them. This means that people may interpret and respond to situations in unique ways based on their personal experiences, beliefs, and values. Prioritization is a reflection of these individual differences in psychological perception. People prioritize different aspects of their lives based on their overall outlook on the world and their personal priorities, leading to a diverse range of priorities and values among individuals.

Differential leaders usually carry out different management behaviors derived from factors such as the cordiality of their relationship, employee loyalty, and the level of job competence, meaning they will behave toward employees differently (Riaz et al., 2018). Psychological contract theory supposes that people have a natural security instinct and desire to be seen as useful, needed, recognized, and valued by their organizations. Therefore, employees in the differential relationship network constantly work toward superiority and priority and refrain from being led into a disadvantaged situation. Employees are constantly altering their behavior as a result of their perception of the organization and rationally devoting their time and energy to integrating into the collective and getting approval from the leader (Pratoom, 2018). Lee and Farh (2019) emphasized that an organization with obviously different levels of dynamic atmosphere can trigger employees’ vitality more effectively compared with a placid emotional atmosphere. Individuals conceive of their work in terms of recognizing the objective existence of differential affective relationships in organizational management. Therefore, they work as hard as possible to gain the favor of their leaders. Differentiated leadership can motivate employees to actively integrate into the organization, inspiring enthusiasm and impacting their job performance.

*H1*: Differential leadership positively impacts employee performance.

### The mediating role of perceived organizational support

Eisenberger et al. (2020) proposed the concept of perceived organizational support from a personal point of view, and describes the relationship between employees and the organization. The main content is the comprehensive evaluation from employees on the levels of the relationship between the individual and the organization, which refers to the scope of the attention and concern from organizational leaders; perceived organizational support advocates that leadership’s care is a prerequisite for employees to utilize their values better. Meanwhile, Kurtessis et al. (2015) proposed that perceived organizational support can measure the degree to which the organization can provide employees with job security and problem-solving, which can encourage employees to form an emotional attachment to their organization. In an organization with a solid dynamic and warm environment, employees will actively overcome work obstacles to increase work input, achieve higher work performance, and obtain more vital organizational emotional care. Leaders will constantly alter their differential styles according to the overall performance of employees.

Meanwhile, employees will constantly alter their work behavior and aim for the opportunity to engage in leadership prioritization (Zhang et al., 2021). In a differential cordial organization, the employee’s work status will be affected immensely by the atmosphere of emotional relationships. Differential leadership thus upgrades the dynamics of the organization’s relational network, stimulates employees to respond positively to organizational dynamics, and continually reinforces the individual’s supportive perception of the organizational environment in the employees’ pursuit of organizational integration. Employees tend to position the organization as a personified individual, and the leader is the spokesperson of it, so the perception of organizational support could be equal to the level of the leader’s care for themselves (Kang and Han, 2021). Although the leader seems like the symbol of a corporate image, as a relatively independent individual, there is a differential feature in handling interpersonal relationships. Leaders are not immune to the impact of cordial relationships on their work. According to the psychological contract theory, employees will be attentive to uncertainties reliant on the organizational situation and caused by direct or continuous actual problems. In most cases, indecisiveness about “who am I” and “what should I do” will cause employees to take corresponding actions to reduce their sense of expectations in the working conditions. At the same time, they may reduce it in the process of positioning and classifying their active roles with other members and increase perceived organizational support through integration into the organization actively, thereby enhancing their organizational identities (Eisenberger et al., 2020). When employees develop a sense of nurturance toward the organization, they will have high levels of affective and normative dedication to their work (Zhu and Guo, 2021). Perceived organizational support may create expectations of increasing returns on investment for employees within a differential leadership organization. The greater the amount of effort employees expend on the organization, the greater the rewards employees receive from the organization (Birze et al., 2022). As a result of the above analysis, the following hypothesis is proposed.

*H2*: Differential leadership positively affects employee performance by improving perceived organizational support.

### The moderating role of individual–organizational fit

Individual–organizational fit reflects the individual’s overall assessment and feedback about the organization. Staff’s behavior toward leadership and their work status is not only dependent on themselves but also on the organizational environment and their relationships (Robertson and Veres, 2022). In addition, Liu et al. (2022) mentions that the relational atmosphere in an organization is the fundamental factor affecting employees’ job performance. For the differential cordial organization with frequently changing relational networks, the introduction of person-organization fit theory can further explain the behavioral level of employees and organizations from two perspectives: constancy and complementarity. Personality reflects essential employee and organizational characteristics such as developmental vision, value orientation, etc. The concept of complementarity means that at least one of the employees and the organization can satisfy the needs of the other, such as through individual competencies and knowledge capabilities (Allen et al., 2022). Kristof et al. (2005) conducted a meta-analysis of 98 studies from Europe, Asia, and North America. They emphasized that individual–organizational fit varies in the organization of Western individualistic culture and Eastern collectivist culture. It is even more critical in the context of Eastern collectivist culture. Chuang et al. (2015) pointed out in a study on individual–organizational fit in Chinese culture that Chinese organizations emphasize harmony in organizational relationships and consider working relationships as the basis for accomplishing their responsibilities to the organization. Individual–organizational fit derives from employees’ perceived recognition of their leaders. A high level of fit means that employees are often acknowledged and cared for by their leaders.

Psychological contract theory suggests that the psychological distance between employees and leaders will foster their enthusiasm for the job (Kutaula et al., 2019). Employees in a low-fit state will aim to improve their workability and performance and take the lead in improving their relationship with leaders in order to achieve emotional care (Eisenberger et al., 2020). Combining the logical mechanism of H1, employees will continue to make alterations to improve work performance to be able to handle the dynamics of the organizational relationship network caused by differential leadership. The high level of individual organizational fit means that employees’ emotional dependency is on the whole organization rather than on a certain leader (Robertson and Veres, 2022). As a result, the higher the personal organizational fit, the fewer employees care about whether or not the leader cares about them. For employees with a high organizational fit, it is difficult for differential leadership to play an effective role in promoting their work, and can even be counterproductive. Thus, the lower the degree of individual-organizational fit, the more effort employees will exert in their work in order to self-adjust and increase their work input. Therefore, the individual-organizational fit will weaken the influence of differential leadership on employees’ job performance. This leads to the following hypothesis.

*H3*: Individual-organizational fit weakens the promoting effect of differential leadership on employee performance.

Perceived organizational support is a social–emotional interaction grounded in leadership between employees and organizations (Hajiali et al., 2022). Once the employees receive emotional care from the leaders, they will fall into the trap of working comfort and become tired of working struggle (Birze et al., 2022). People do not often engage in “thinking of danger in times of safety” (Jeong and Kim, 2021). According to psychological contract theory, the higher the individual-organizational fit, the more organizational support is perceived by differential leadership as their capital for job stability (Chuang et al., 2015). Employees will begin to stray away from taking the initiative to increase their work effort. Conversely, the weaker the individual-organizational fit, the more employees will strive to improve their performance to gain a sense of belonging. As a result, the effect of perceived organizational support on employee performance is more potent in conditions of low individual organizational fit (Robertson and Veres, 2022). This occurs when employees gain a sense of satisfaction with certain social and emotional resources in their vicinity, which can then lead them to want to continue to recognize the organizational environment and to actively adapt to the organizational environment. Employees will be stimulated to understand leadership style and organizational culture and to respond to team spirit and shared values. Through this process, the constructs of perceived organizational support and individual-organizational fit could work together, reflecting employees’ internal perceptions of organizational characteristics (Langley et al., 2009). Leaders and employees evaluate their expectations of each other and then adjust their respective perceptions based on the level of congruence between expectations and actual performance. And the level of congruence is the deterministic variable of individual–organizational fit. A lower individual-organizational fit means there is more room for both parties to change their behavior toward each other (Coyle-Shapiro et al., 2019). Therefore, a higher private organization fit means that the actual behavioral performance is more consistent with expectations, and there is less room for adjustment of attitude.

Employees exceeding expectations often cause a boost in employee performance. Superior performance requires frequent breakthroughs in work practices and breaking leadership preconceptions (Sorlie et al., 2022). However, employees that have close relationships with their leaders tend to develop work inertia. Only employees distant from the leader are motivated to work to gain the leader’s favor (Chen and Haga, 2022). The lower the individual-organizational fit, the stronger the motivational effect of perceived organizational support on employee performance, and vice versa (Offermann and Coats, 2018). Therefore, in combination with the intermediary transmission mechanism of H3, in a highly dynamic organization with a sturdy network of emotional relationships, the effect of individual–organizational fit could throw off the impact of perceived organizational support on employee performance. Based on this, the following hypothesis is proposed.

*H4*: In a differential cordiality organization, individual organizational fit weakens the promoting effect of perceived organizational support on employee performance.

**Figure 1:**
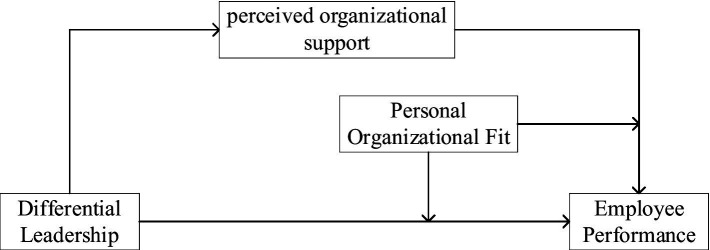
Theoretical model.

In summary, the content explored in this paper is the process of conditioning differential leadership on employee innovation behavior, which involves the mediating variable of perceived organizational support and the moderating variable of individual–organizational fit. The structure of the theoretical model is shown in Figure 1.

## Research design

### Sample and procedure

Our method of gathering data was through questionnaires. The questionnaire collected data from 57 companies whose primary businesses are equipment manufacturing, information technology, and daily chemical production. The types of enterprises include state-owned, private, and mixed-ownership enterprises. After receiving the company’s permission, a selection of 450 employees were chosen to participate in the survey at random. With the cooperation of the personnel department, electronic survey questionnaires were distributed from all corners through the company’s internal platform. Members of the research team provided on-site guidance and answered questions. After the questionnaires had been completed, we expressed our gratitude to each respondent by providing them with a coupon. Leaders answered questions about employee performance and control variables. Employees answered questions on differential leadership, perceived organizational support, and individual–organizational fit. We matched leaders with employees through questionnaire coding. The investigation lasted nearly 4 months.

After eliminating unusable questionnaires such as those with inconsistent answers, incomplete answers to questions, or missing core information, 358 valid questionnaires were obtained. The research population included 223 male and 135 female respondents. There ages ranged from 26 to 44 years old. All were in possession of a bachelor’s degree or higher, and their average work experience was 4.6 years. Among them, 142 respondents were from state-owned equipment manufacturing enterprises and their subsidiaries, 95 from private information technology enterprises, and 121 from joint venture daily chemical enterprises. Their positions include professional and technical, middle management, marketing, and administrative services. The obtained survey information satisfies the requirements of the empirical analysis in this paper.

### Measures

The scales used in this paper are authoritative mature scales, with high reliability and validity. All questionnaires are designed with a Likert 7-point scale, with 1 (completely disagree) to 7 (completely agree) to indicate the respondent’s evaluation of the listed items.

### Differential leadership

Most previous papers measuring differential leadership have used leader or team leader self-evaluation approaches, which are prone to covariance bias (Xu et al., 2022). Gathering answers from employees circumvents the covariance problem arising from leader self-evaluation. We referenced and incorporated scales used by most researchers (Lu et al., 2022). This scale consists of 12 question items. The contents of the questions focus on asking employees to determine whether leaders tend to take more care of employees who are close to them. The Chinese version of our questionnaire has high internal consistency, reliability, and good content validity to meet the requirements of this paper. The Cronbach’s alpha coefficient for the Chinese version of the questionnaire was 0.906.

### Perceived organizational support

The questionnaire used to measure perceived organizational support in China was derived from foreign literature (Kurtessis et al., 2015). For foreign scales, the nonprofessional international scales will be first translated and then modified by expert teachers to avoid semantic deviation. The scale widely used in academia was developed by Eisenberger et al. (2020) and later optimized several times. The result is a scale that includes eight items. The contents of the questions cover the leaders’ concern in caring about employees’ welfare, whether leaders can help employees with difficulties promptly, and whether leaders pay attention to employees’ individual needs (Griep and Bankins, 2022). The consistency coefficient of this scale was 0.907.

### Individual–organizational fit

There are two types of views for understanding individual–organizational fit: the “material view” and the “spiritual view.” The “material view” interprets personal and organizational fit from the perspective of benefit exchange and believes that the degree of satisfaction with the organization’s benefits directly determines the degree of individual and organizational fit (Robertson and Veres, 2022). The “spiritual view” focuses on employees’ emotional relationship with their leaders and the organization. It believes the strength of employees’ sense of belonging to the organization is the decisive factor of individual–organizational fit (Liang et al., 2022). This paper uses a questionnaire developed by Sahlmueller et al. (2022). This questionnaire includes four questions on personal values and organizational development vision, leaders’ attitudes toward employees, organizational integrity philosophy, and organizational cooperation climate. The consistency coefficient of the items included in this questionnaire is 0.781, which meets the research requirements.

### Employee performance

Employee performance includes explicit organizational assessment indicators and implicit work status (Kutaula et al., 2019). To some extent, implicit job status is the decisive factor that affects employee performance (Sorlie et al., 2022). As a result, this paper uses questionnaires to scale employee performance. It should be noted that the leader fills out this questionnaire to avoid bias in the results generated by employee self-evaluation. The researchers asked the leaders to measure employee performance based on their work results and daily work status. The questionnaire consists of four items: task completion, team adaptability, interpersonal relationship, and work effort (Haibo, 2022). The consistency coefficient is 0.730 upon examination, which has good structural testability and meets the research requirements of this paper.

### Control variables

To test the net effect of differential leadership on employee performance, we controlled for several factors in our hypothesis analysis that might confound the relationship between the core variables in this paper. These factors include several demographic characteristics of leaders and employees, such as gender, age, education, and work experience. Referring to the literature on leadership-type research, we further controlled for variables such as job position, team size, and company type (Jeong and Kim, 2021). The purpose is to ensure that the results of our data analysis reflect the proper relationship between the core variables.

## Data analysis and results

### Confirmatory factor analysis

To test the discriminant and convergent validity of all the conceptions in our model, a validated factor analysis was conducted on four concepts with the Mplus Version 8.3: differential leadership, perceived organizational support, individual–organizational fit, and employee performance. The results are shown in Table 1. The four-factor model [(*χ*^2^/*df* = 2.073) < 3, RMSEA = 0.038, SRMR = 0.047, CFI = 0.934, TLI = 0.938] is significantly better than other factor combination models, indicating that the four variables concerned in this paper have good convergent validity and discriminative validity. This result is consistent with the expectations of psychological contract theory. Leadership type affects employee perceptions of organizational support and employee performance (Robertson and Veres, 2022), and these influence relationships are subject to personal and organizational fit (Lerutla and Steyn, 2022).

**Table 1:** Confirmatory factor analysis.

Models	*χ* ^2^	*df*	*χ*^2^/*df*	RMSEA	SRMR	CFI	TLI
Four factors model (DL, POS, POF, EP)	659.074	318	2.073	0.038	0.047	0.934	0.938
Three factors model1 (DL + POF, POS, EP)	1030.785	321	3.211	0.073	0.082	0.801	0.783
Three factors model2 (DL,POS + POF, EP)	1108.082	321	3.452	0.075	0.083	0.787	0.767
Three factors model3 (DL + POS, POF, EP)	1186.069	321	3.695	0.082	0.087	0.748	0.724
Two factors model (DL + POS + POF, EP)	1321.576	323	4.092	0.089	0.090	0.701	0.675
One factor model (DL + POS + POF + EP)	1698.581	324	5.243	0.105	0.096	0.654	0.625

DL = differential leadership, POS = perceived organizational support, POF = individual-organizational fit, EP = Employee Performance. The symbol “+” means combining variables.

### Common method deviation test

Despite our best efforts to refine data collection methods, common methodological biases may exist because employees provide data through self-reported methods in collecting information. With reference to established studies (Langley et al., 2009), it can be controlled from two aspects, namely survey operation procedures and statistical inspection methods, which can test the common method variance problem. Looking at the actual situation of this paper, the following surveys are the methods used in process adoption. The first one is to optimize the structure of the questionnaire and adjust the order of variable entries to minimize the problems of homologous deviation, such as balanced responses to the questionnaire and answers that have already guessed the purpose of the survey (Su and Sun, 2020). The second is to think over the expression of variable questions, to use neutral words as much as possible, and design reverse questions to avoid consistent answers as much as possible.

Building upon the work of Fang et al. (2018), we employed the single factor experimental method suggested by Harman to evaluate the potential presence of common method bias in our model. Our findings indicate that the factor with an eigenvalue greater than 1 explained 76.49% of the total variance, which accounted for more than half of the total variance. Additionally, the first factor accounted for 20.81% of the total variance, which is less than 40%. Based on these results, we can conclude that any common methodological bias in our research data is not significant.

### Descriptive statistics and correlation analysis

The correlation test measures the relationship between the conceptions in the model and their significance. As shown in Table 2, the correlation coefficient between differential leadership and employee performance (*r* = 0.586, *p* < 0.001) indicates a significant positive correlation between them. This is the same as perceived organizational support (*r* = 0.509, *p* < 0.001). Based on the correlation coefficients and significance results between perceived organizational support and employee performance (*r* = 0.609, *p* < 0.001), there is a mutually reinforcing relationship between them. This result reflects the findings of Jeong and Kim (2021). Individual–organizational fit has a positive connection with both employee performance (*r* = 0.631, *p* < 0.01) and perceived organizational support (*r* = 0.323, *p* < 0.01). The above results indicate a correlation between the core conceptions in the theoretical model. The next step of empirical analysis can be performed.

**Table 2:** Means, standard deviations, and correlation coefficients of variables.

	*M*	SD	1	2	3	4	5	6	7	8
1. Post	3.21	0.955	1							
2. Nature of enterprise	1.87	0.776	0.011	1						
3. Length of service	1.96	0.415	0.007	0.045^*^	1					
4. Team size	3.01	1.004	−0.016	−0.039	−0.047	1				
5. Differential leadership	2.914	0.056	0.019	0.072^*^	0.067	0.091^*^	1			
6. Perceived organizational support	2.944	0.041	0.046^*^	0.107^*^	0.043	0.043^*^	0.509^***^	1		
7. Individual-organizational fit	3.246	0.056	−0.016	0.048	0.091^*^	0.011	0.434^**^	0.323^**^	1	
8. Employee performance	2.769	0.051	−0.006	0.132^*^	0.086^*^	0.024	0.586^***^	0.609^***^	0.631^***^	1

^*^p < 0.05, ^**^p < 0.01, ^***^p < 0.001.

### Hypotheses test

Mplus8.3 was used for hierarchical regression analysis in this study to test the hypotheses of the overall sample, including 358 valid questionnaires from 57 companies.

### Mediating effect

**Figure 2:**
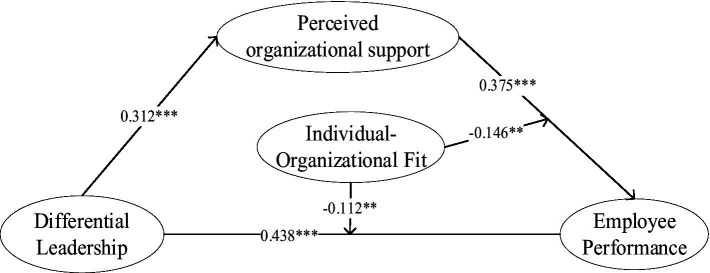
Hypothesis testing results. ^*^p < 0.05, ^**^p < 0.01, ^***^p < 0.001.

The organizational behavior of differential leadership is mainly attributed to the cordial leader-employee relationship (Lee and Farh, 2019). The degree of emotional cordiality directly affects the leader’s attitude toward employees. We used structural equation modeling to test our theoretical hypotheses, and the results are shown in Figure 2. Empirical results support all the study’s theoretical hypotheses.

To ultimately show the results of the empirical analysis, we present all the resultant data in Table 3. Hypothesis 1 advocates that differential leadership impacts employee performance through perceived organizational support. The test results are shown in Table 3. After controlling for variables such as job position, length of service, team size, and the nature of the enterprise, differential leadership positively impacts perceived organizational support (0.312; 95% CI [0.357, 0.562]) and employee performance (0.438; 95% CI [0.316, 0.561]), which indicates that the perceived organizational support plays a mediating role. Therefore, H1 and H2 are not negated.

**Table 3:** Bootstrapping results for mediation effect.

	Perceived organizational support(POS)					Employee performance(EP)				
	Coeff	se	*t*	LLCI	ULCI	Coeff	se	*t*	LLCI	ULCI
*Main effects*										
DL	0.312	0.052	8.798^***^	0.357	0.562	0.438	0.062	7.045^***^	0.316	0.561
POS						0.375	0.058	7.498^***^	0.319	0.545
*Controls*										
gend	0.067	0.077	0.871	−0.084	0.217	0.069	0.083	0.830	−0.094	0.231
age	−0.031	0.039	−0.815^*^	0.107	0.244	0.042	0.042	1.012^**^	0.040	0.124
post	0.013	0.037	0.357	−0.060	0.087	−0.023	0.040	−0.577	−0.102	0.056
bcap	0.048	0.046	1.029^**^	0.043	0.138	0.079	0.050	1.586^*^	0.019	0.177
seni	−0.023	0.086	−0.267	−0.191	0.145	−0.118	0.092	−1.285	−0.300	0.063
scale	−0.012	0.038	−0.306	−0.085	0.062	−0.017	0.040	−0.409	−0.096	0.063

*N* = 358. DL = differential leadership, LLCI = lower limit confidence interval, ULCI = upper limit confidence interval. ^***^, ^**^, ^*^ indicate *p* < 0.001, *p* < 0.01, *p* < 0.05.

### Moderating effect

Personal organizational fit is closely related to the idea of employee engagement, which is the degree of commitment and loyalty a worker has to their leaders. Hypothesis 3 argues that the impact of differential leadership on employee performance is regulated by individual–organizational fit. The results of the bootstrapping are shown in Table 4. Individual–organizational fit has a negative regulatory impact on the main effect (−0.112; 95% CI [−0.221, −0.003]), that is, the individual–organizational fit would weaken the impact of differential leadership on employee performance. Hypothesis 3 has been verified. Hypothesis 4 advocates that the individual-organizational fit negatively regulates the impact of perceived organizational support on employee performance. The results in Table 4 verify that the negative moderating impact of the individual–organizational fit in the mediation model is significant (−0.146; 95% CI [−0.252, −0.039]), and the research Hypothesis 4 is not rejected.

**Table 4:** Bootstrapping results for moderated mediating effect.

	Employee performance(EP)					Employee performance(EP)				
	Coeff	se	*t*	LLCI	ULCI	Coeff	se	*t*	LLCI	ULCI
*Main effects*										
DL	0.287	0.060	4.794^***^	0.169	0.405	0.284	0.060	4.772^***^	0.167	0.401
POF	0.380	0.050	7.622^***^	0.282	0.479	0.367	0.053	6.911^***^	0.263	0.472
POS	0.376	0.053	7.063^***^	0.271	0.480	0.398	0.049	8.117^***^	0.301	0.494
*Interaction effects*										
DL × POF	−0.112	0.056	−2.014^**^	−0.221	−0.003					
POS × POF						−0.146	0.054	−2.691^**^	−0.252	−0.039
*Controls*										
gend	−0.004	0.076	−0.045	−0.153	0.146	−0.001	0.076	−0.018	−0.151	0.148
age	0.022	0.038	0.575^*^	0.053	0.097	0.025	0.038	0.667^**^	0.049	0.100
post	−0.009	0.037	−0.256	−0.082	0.063	−0.011	0.037	−0.309	−0.083	0.061
bcap	0.076	0.046	1.657^**^	0.014	0.165	0.073	0.045	1.606^**^	0.016	0.162
seni	−0.094	0.085	−1.115	−0.261	0.072	−0.100	0.084	−1.183	−0.266	0.066
scale	−0.008	0.037	−0.205	−0.081	0.066	−0.007	0.037	0.177^**^	0.079	0.166

*N* = 358. LLCI = lower limit confidence interval, ULCI = upper limit confidence interval. ^***^, ^**^, ^*^ indicates *p* < 0.001, *p* < 0.01, *p* < 0.05.

**Figure 3:**
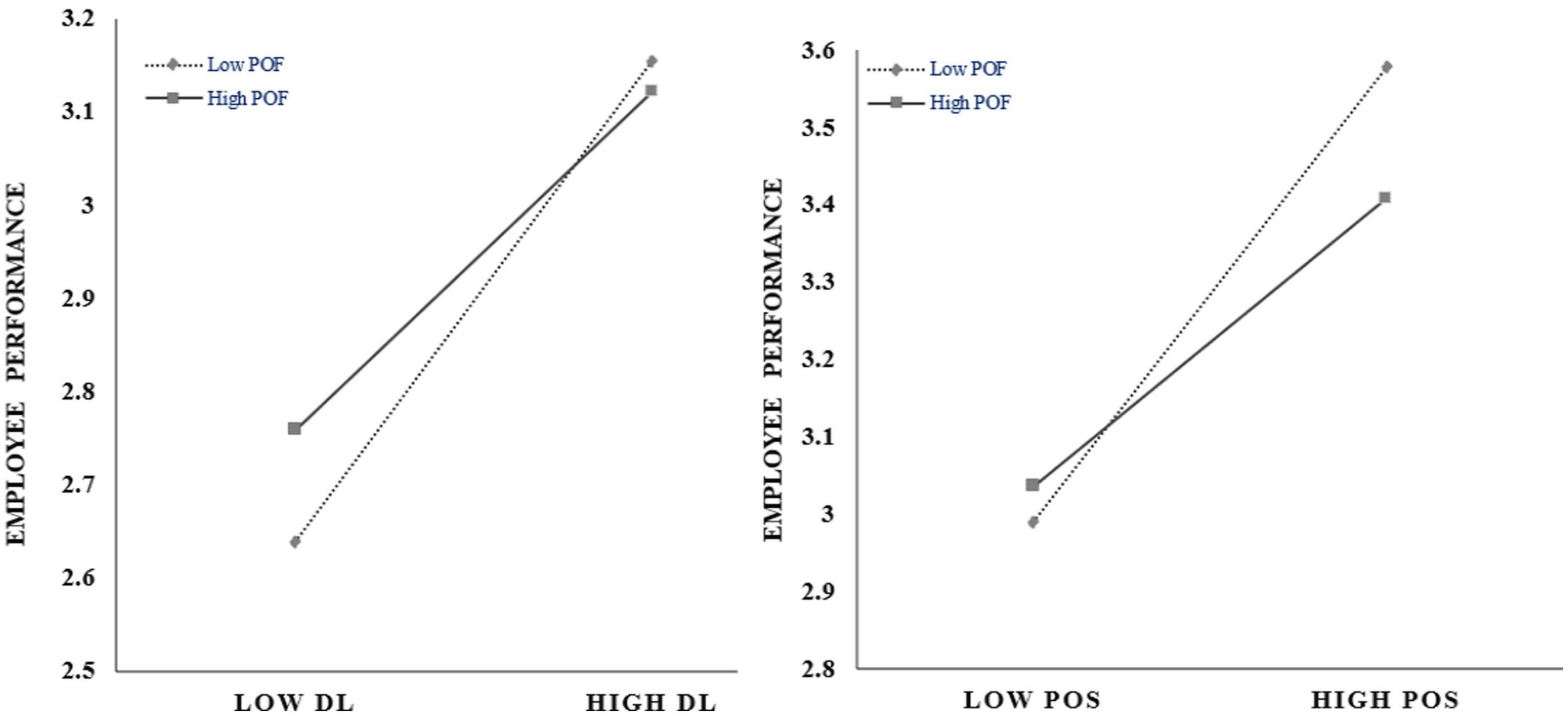
Plots for the interaction effects.

Furthermore, we examined the indirect effects of the two interaction conditions described earlier. When the individual–organizational fit is low, the differential leadership has a significant impact on employee performance, with an index of (0.373, 95% CI [0.237, 0.509]); when the individual–organizational fit is high, the impact effect is also highly significant, but the degree of impact has decreased (0.201; 95% CI [0.048, 0.354]). The individual-organizational fit weakens the impact of differential leadership on employee performance (Figure 3), which supports Hypothesis 2. At the same time, when the individual–organizational fit is low, the differential leadership significantly positively impacts performance through the perceived organizational support; the parameter is (0.480, 95% CI [0.353, 0.606]). When the individual-organizational fit is high, the degree of this indirect impact decreases (0.255; 95% CI [0.116, 0.394]), supporting H3 (Figure 3). What needs to be emphasized is that the data is standardized in the empirical process of this paper. Our analytical model explains 23% of the variance in employee performance, which is a high level of explanation.

## Conclusion and discussion

The paper focuses on the impact of interpersonal cordiality on employees’ job performance. Based on psychological contract theory, this paper conducts an exploration of how cordiality between employees and leaders affects employee performance. Through empirical analysis, this paper draws several conclusions: (1) Differential leadership does motivate employees and improves their performance. The interpersonal closeness between leaders and employees becomes a core variable influencing leadership behavior (Su and Sun, 2020). Differential leadership only favors employees who work hard (Wang et al., 2018). So, the only way for employees to gain leadership preference is to work actively and achieve a certain level of job performance; (2) Differential leadership enhances employees’ perceptions of organizational support, and further improves employee performance through it. The perception of organizational support is a fundamental condition for employees to form organizational identity and organizational belonging (Zhang et al., 2021). This emotional satisfaction can make employees take the initiative to increase work commitment and actively complete the work tasks assigned by the leadership; (3) Individual organizational fit can diminish the positive effects of differential leadership on employee performance. Employees see building close interpersonal relationships with their leaders as the ultimate goal of their work (Liu et al., 2022). Once employees have gained the favor of their leaders, they will lose the motivation to continue working hard; and (4) Individual organizational fit also weakens the positive effect of perceived organizational support on employee performance. In other words, many factors play a positive role in employee performance, but the positive effects of differential leadership and perceived organizational support are diluted by individual organizational fit. These findings expand the understanding of differential leadership and also serve as a practical guide.

There are still some controversies about how differential leadership affects organizations and employees. In the framework of psychological contract theory, differential leadership with Chinese cultural characteristics has a certain effect on employee performance. Our findings provide several new empirical and theoretical contributions as follows.

### Theoretical implications

Differential leadership is a leadership style based on Chinese relational (guanxi) culture. We discussed the origins of differential leadership and pointed out that its impact on employees is a continuous and dynamic process. Previous studies have divided the management objectives of differential leadership into “insiders” and “outsiders” (Nassif et al., 2021). This is inconsistent with the essence of the differential relationship because it is not an antagonistic relationship but refers to the dynamic process of interpersonal relationships. The differential cordiality schemas, proposed by the Chinese sociologist Fei Xiaotong, refer to the process of interpersonal relationship change. The differential cordiality schemas describe the “differentiation” and “prioritization” characteristics of interpersonal cordiality in organizations (Chong, 2022). When the focused area of research on differential leadership is restricted to discussing the issues of “outsiders” and “insiders,” researchers will be limited to discussing how to resolve interpersonal conflicts instead of the interpersonal beneficial interactions. As a result, academics will only see the negative effects of differential leadership without a deep understanding of its positive effects. It is important to note that the effects of differential leadership are not only limited to the “insiders,” but also to all the members who work within it. Lopez-Cabarcos et al. (2021) pointed out that employees will gradually move closer to the center of the differential cordiality schema even if they are in a bad situation. Therefore, employees should not be arbitrarily divided into “insiders” and “outsiders,” but all employees should be regarded as “family members” in the process of studying differential leadership. The above theoretical account of differential leadership is consistent with the original thinking of differential cordiality schemas.

In addition, we reveal the action mechanism of how differential leadership affects employee performance based on the psychological contract theory. Psychological contract theory emphasizes the implicit emotional identity between leaders and employees as the core variable influencing the relationship within the organization (Offermann and Coats, 2018). A psychological contract is a set of intangible, implicit, and unwritten expectations that are embedded in the interpersonal relationships between leaders and employees. Differentiated leaders always adopt unique emotional ways to communicate with employees and build mutual trust by increasing emotional care for employees (Davidescu et al., 2020). If employees enjoy the priority differential of their leader and maintain a close relationship with the leader, a strong emotional attachment to leadership is formed, which reinforces employees’ perception of organizational support and further stimulates their sense of work efficacy (Chen and Haga, 2022). Perceived organizational support and individual organizational fit are the core variables psychological contract theory addresses. The research findings of this paper suggest that differential leadership is one way to enhance employee performance by increasing employees’ perceived organizational support. The degree of individual organizational fit determines the degree of stability of the employee-leader partnership. Sahlmueller et al. (2022) believes that only when the employee-organizational fit is at a certain level can the synergistic relationship continue. Therefore, the relationship between differential leadership and employee performance is restricted by the level of individual organizational fit. The relationship between these implicit organizational psychology variables has received less attention in previous research literature.

Furthermore, the paper extends the application scenarios of psychological contract theory. Our findings suggest that leadership behavior is influenced not only by individual psychological factors but also by sociocultural context. Not only is the interpersonal psychological contract built on the exchange of material benefits, but it is also built on the satisfaction of the spiritual dimension (Kutaula et al., 2019). Psychological contract theory stresses that implicit interpersonal psychological identity requires overcoming the uncertainty of the external world (Soares and Mosquera, 2019). The effects of differential leadership on employees are concentrated in the features of dynamic changes in the leadership-employee relationship. Workers have an innate enthusiasm to reduce uncertainty about the environment around them and to actively integrate themselves into the organization, i.e., they typically do not spontaneously reduce their perceived organizational support (Robertson and Veres, 2022). In organizations with ever-changing cordial interpersonal relationships, employees’ perceived organizational support could be strengthened. Psychological contract theory points out that there will be preconceived assumptions between leaders and employees (Jeong and Kim, 2021). People determine how to treat others based on judgments of the fit between preconceived hypotheses and the actual image (Coyle-Shapiro et al., 2019). Thus, the degree of fit between leaders and employees has a direct bearing on the stability of their working relationship. This is also the key to psychological contract stability. Individual-organizational fit reflects consistency in both individual and organizational goals (Chuang et al., 2015). Collectivism and individualism are also one of the differences between East Asian culture and European culture, which pervade business organizations and affect individual organizational behavior (Chong, 2022). Zhu and Guo (2021) points out that person-organization fit has a positive impact on employees’ performance in an individualistic organization, but this is not true in collectivistic organizations. In contrast, cordiality in interpersonal relationships is the fundamental variable that impacts employee behavior in a collectivist organization. The empirical results of this paper suggest that individual–organizational fit plays a negative moderating role in the model. It represents, to some extent, the characteristics of organizations and employees within the context of Chinese culture.

### Managerial implications

The performance of employees is affected not only by individual psychological factors but also by cultural features of social relations (Hajiali et al., 2022). It is critical to recognize that the degree of interpersonal cordiality affects the state of work between leaders and employees. Given that differential leadership can have a positive impact on employee performance, it implies that the overall work performance of the firm can be enhanced by expanding the coverage of leadership relationships. Establishing close interpersonal relationships has become one of the means of improving employee performance. A practical guideline that can be derived is that “relationships (guangxi) are the motivation that drives employees to work.”

Perceived organizational support is a precondition for the formation of a stable psychological contract between employees and leaders. The empirical findings of this study suggest that differential leadership may provide employees with perceptions of organizational support (Bush et al., 2021). On the one hand, there is a need to open the channels of communication between leaders and employees so that employees can perceive leaders’ affectivity by increasing the coverage of leaders’ interpersonal networks (Haibo, 2022). On the other hand, work relations and interpersonal relations are inseparable from organizational relations. As Lerutla and Steyn (2022) has pointed out, mutual support between leaders and employees is one of the conditions guaranteed to improve organizational performance. People, be they employees or leaders, need organizational assistance to motivate their work. As a result, there is more encouragement and less blame between leaders and employees in order to create an excellent organizational atmosphere.

We found an interesting finding that individual organizational fit decreases the positive impact of differential leadership on employee performance. This negative moderating effect of individual–organizational fit releases a “warning” signal for the organizational management practice of differential leadership. Individual–organizational fit is a psychological perception of employees based on their emotional experience, reflecting their sense of embeddedness and belonging within the organization (Kang and Han, 2021). At present, organizational fairness is regarded as the primary criterion of environmental rationality, and the differentiated leadership approach runs counter to the criterion of fairness. Given that it is difficult to maintain a high level of individual-organizational fit for employees in a messy organization, differential leaders must be flexible in adjusting their disorder management behavior toward employees. The cordial relations between leaders and employees must not be too close or too distant. An appropriate degree of cordiality effectively motivates employees’ enthusiasm for work. This may be the epitome of relational culture with Chinese characteristics in organizational management.

### Limitations and prospects

We explain how interpersonal cordiality affects leader-employee organizational behavior. Differential leadership constructs provide the research script as this is a type of leadership based on relational cordiality. Even though we have found a better search scenario to solve the search problem, this paper still has a few things that could be improved. First, the variable measurement approach we adopt is the cross-rater approach to avoid common method bias. Employee performance, for example, is assessed by leaders. Future studies could use mutual assessment between colleagues at the same level. Second, our data is from Chinese firms only, which limits the generalizability of the findings of this paper. Thirdly, differentiated leadership is a leadership style unique to China, originating in Chinese interpersonal culture, and difficult to articulate its essential connotation in other linguistic environments. These aspects can be further explored in follow-up studies.

## Data availability statement

The original contributions presented in the study are included in the article/supplementary material, further inquiries can be directed to the corresponding author.

## Author contributions

NL contributed to the empirical methodology and data curation and wrote the manuscript. HZ developed the theoretical formalism and supervised the work. JZ contributed to data and literature collection. All authors have read and agreed to the published version of the manuscript.

## Funding

The National Natural Science Foundation of China “Study on the Causes, Evolution and Resolution Mechanism of Ought to be Conflicts in Mixed Ownership Reform: The Perspective of a mutually beneficial organization (No. 72262035)”; the Scientific Research Fund Project of the Yunnan Education Department “Measurement and influencing factors of green innovation efficiency of state-owned enterprises in Yunnan Province under the background of high-quality economic development (No. 2022Y482)”; and the Guizhou Education Science Planning Project “Research on the Internationalization Development Path of Guizhou Higher Vocational Education under the Background of Upgrading of the Inland Open Economy Pilot Zone Construction (No. 2022C042)”.

## Conflict of interest

The authors declare that the research was conducted in the absence of any commercial or financial relationships that could be construed as a potential conflict of interest.

## Publisher’s note

All claims expressed in this article are solely those of the authors and do not necessarily represent those of their affiliated organizations, or those of the publisher, the editors and the reviewers. Any product that may be evaluated in this article, or claim that may be made by its manufacturer, is not guaranteed or endorsed by the publisher.
